# The Impact of *Clinacanthus nutans* (Burm. F.) Leaf Extract on Sperm Quality and Antioxidant Activity in Male Mice Induced with Streptozotocin

**DOI:** 10.5935/1518-0557.20240010

**Published:** 2024

**Authors:** Samiaa Jamil Abdulwahid-Kurdi

**Affiliations:** 1 General Sciences Department, Faculty of Education, Soran University, Soran, Erbil Kurdistan Region of Iraq

**Keywords:** antioxidant, Clinacanthus nutans, infertility, sperm motion, streptozotocin

## Abstract

**Objective:**

*Clinacanthus nutans* (*C. nutans*) is a medicinal herb that most people with diabetes have historically taken. It’s a diet high in antioxidants, which are supposed to help people live longer and be healthier. It is the first study to suggest using *C. nutans* to enhance the quality of sperm in male mice given a streptozotocin (STZ) injection.

**Methods:**

Sixty mice were divided into two groups at the age of four weeks: group one was fed a regular diet (n=10), while group two was administered a high-fat diet (n=50) for eight weeks to develop obesity. Obese mice were given 100mg/kg of STZ to produce hyperglycemia with a 20% mortality rate. Then, 40 hyperglycemic mice were separated into two groups: STZ (n=10) and sample (n=30). The treatment groups were administered a methanolic extract of *C. nutans* leaves by gavage at doses of 150, 300, and 500mg/kg of body weight (n=10) for 4 weeks.

**Results:**

In contrast to the STZ group, there was a substantial (*p*=0.001) drop in serum blood glucose and total sperm abnormalities in mice at varying doses. Catalase, glutathione s-transferase (GST), and total antioxidant capacity significantly (*p*=0.001) increased in the STZ mice group at varying doses, but malondialdehyde was reduced. In comparison to STZ mice, testosterone and luteinizing hormone (LH) levels improved in mice treated with extracts of *C. nutans* at various doses. For all of the following dependent variables, extraction of the leaf at higher concentrations of 500 milligrams/kilogram has better efficacy than 300 and 150 mg/kg after 4 weeks of treatment.

**Conclusions:**

The research and development of new natural agents to combat oxidative stress-related diseases have sparked a lot of interest. As a result, the potential leaf extract of *C. nutans* contains anti-hyperglycemic compounds and improves the quality of sperm in male mice.

## INTRODUCTION

A disorder of the reproductive system known as infertility is defined as the inability to conceive clinically after 12 months or more of frequent, unprotected sexual activity (Szamatowicz & Szamatowicz, 2020). According to estimates from the World Health Organization (2023), infertility affects 1 in 6 persons, or 17.5% of the world’s population. A cycle of in vitro fertilization (IVF) is charged in the range of USD 12000 and USD 17000, with some reaching up to USD 25000 or higher (Klein, 2020). Most of the participants interviewed (46%) have public health insurance, not including the costs of fertility-related problems (Klein, 2020). The majority of people seeking infertility treatment with private insurance, on the other hand, were equally anxious. Meanwhile, IVF is costly and has socio-economic problems that may be due to multiple births (Klein, 2020). Male-factor infertility affects over half of infertile couples, with just 30% of those couples experiencing exclusively male-factor infertility (Reed-Maldonado & Madden, 2019). Both type 1 and type 2 diabetes have a detrimental effect on sperm maturation, hormonal alterations, and testicular function in the male reproductive system (Sasaki & Matsui, 2008). Particularly in terms of sperm quality, motility, DNA integrity, and seminal plasma components (Sasaki & Matsui, 2008). According to the International Diabetic Federation (2019), around 463 million adults worldwide had diabetes in 2019, with the majority living in lowand middle-income countries; by 2045, this figure is anticipated to rise to 700 million. According to Nak-ung *et al*. (2018), diabetes mellitus (DM) is a metabolic disorder characterized by poor protein, lipid, and glucose metabolism. Due to their cumulative long-term harm, free radicals have been linked to a number of degenerative diseases and maladies (Temple, 2000).

There is evidence that several plant-based polyphenols have the ability to neutralize free radicals and have anti-inflammatory, hypoglycemic, and anti-infertility effects (Karale *et al*., 2020). With a range of side effects and drug resistance, herbal therapy may be a viable alternative. Plants are increasing in widespread popularity as a source of medication due to their natural origins, availability in local communities, cheaper purchase costs, ease of administration, and maybe fewer bothersome side effects (Sasidharan *et al*., 2011). Several plants have been demonstrated to be useful in treating various aspects of male infertility (Nantia *et al*., 2009). Another option is *Clinacanthus nutans* (*C. nutans*) Lindau, belonging to the family Acanthaceae, which is well-known in Malaysia as Sabah snake grass. Since the rising demand for plant-based drugs increases the risk of adulteration or substitution, plant species, and phytoconstituent are required for effective treatments (Ilacqua *et al*., 2018). Phytochemical substances found in *C. nutans*, such as terpenes, alkaloids, protocatechuic acid, phenols, steroids, and tannins, can contain anti-hyperglycemic and anti-hyperlipidemic properties (Abdulwahid-Kurdi, 2019). Traditionally, the leaves of *C. nutans* are sun-dried and steeped as a tea to cure many diseases like diabetes, dysuria, diarrhea, and many other diseases in Southeast Asia, particularly in Malaysia, Indonesia, and Thailand. Nonetheless, more empirical evidence was necessary to support such beliefs. As a result, the findings of this study provide scientific evidence that *C. nutans* has the ability to serve as a fertility agent in an animal study. Streptozotocin (STZ), a glucosamine-nitrosourea chemical compound derived from *Streptomyces achromogens* bacteria, has a wide range of antibacterial properties (Lenzen, 2008). Male ICR mice are more likely to develop hyperglycemia and induce obesity than female mice (Kondo *et al*., 2000; Wang & Liao, 2012). Eating a high-fat diet to generate insulin resistance while injecting low-dose STZ can cause hyperglycemia (Skovsø, 2014). To put it another way, mice are more resistant to the substance than rats. According to King (2012), a high dose of STZ of 100 to 200 mg/kg can produce non-insulin-dependent diabetes mellitus in mice, whereas a dose of 35-65 mg/kg can cause the same result in rats. Hence, the main aim of this research was to determine the effects of *Clinacanthus nutanus* crude leaf extract on blood glucose, sperm activity, and antioxidant enzymes in diabetic male mice.

## MATERIALS AND METHODS

### *Clinacanthus nutans* Crude Methanolic Leaf Extract Preparation

*Clinacanthus nutans*, fresh (Burm. f.) Lindau leaves were obtained from a botanical garden at Field 10 UPM, Selangor, Malaysia. The study takes place between latitude 2.991299 and longitude 101.708182. Phytomedicine Herbarium, Institute of Bioscience, UPM, Selangor (Voucher number. SK2942/15) identified the botany of the *C. nutans*. The extracts were produced using the Abdulwahid-Kurdi (2019) procedure. The leaves of *Clinacanthus nutans* were thoroughly cleaned, and dried for seven days in the shade. Then the leaves are baked for 24 h at 40°C, then processed into a ground texture with an electric grinder (RT-08, Rong Tsong Precision Technology Co. Taiwan) and kept in an airtight plastic rubber. At a ratio of 1:20 (w/v), 1g of material to 20 ml of methanol, ground leaf samples were extracted with 80 percent methanol (20 percent distilled water). Using a rotary shaker, ground *C. nutans* leaves were softened in methanol for 72 h (Liquid Brushless DC motor clock Rotary, Germany). Using cotton, cloth wool, and Whatman No. 1 filter paper (Whatman No. 1, Fitchburg, WI, USA), the methanol solution was removed from the powdered leaves. A spinning evaporator was used to concentrate the methanol extract at 40°C under compact pressure (R-215, Buchi, Flawil, Switzerland). A freeze drier was used to lyophilize the concentrated methanol (Labconco Free zone 6 Plus Freeze Dryer) and kept at -20°C after being concentrated at -80°C. The methanol extract produced 15.92 percent of the total yield (w/w).

### Animal Model

Sapphire Enterprise in Malaysia gave sixty 4-week-old ICR male mice with an average weight of 20.2g and appeared to be in good health. They were provided a standard laboratory mouse diet and had unrestricted water access. All approaches and steps employed in this study were investigated and authorized by the UPM Institutional Animal Care and Use Committee (IACUC) (ACUC Approval No: R083/2016).

### Diet Composition and Induction of Insulin Resistance

After 7 days of acclimating to their new surroundings, the animals were randomly divided into two groups. The first group of 10 mice was fed ordinary chow, while the second group of 50 mice was initially fed a high-fat diet for eight weeks that had around 48% total carbohydrates, 17% protein, 31% fat, 3% fiber, and 5% of a mineral and vitamin combination to produce obesity (Kondo *et al*., 2000). Streptozotocin (STZ) (Sigma, S0130-USA) was newly prepared (100 mg/kg body weight) (Srinivasan & Ramarao, 2007; Dekel *et al.*, 2009), dissolved in sodium citrate buffer, pH 4.5 to induce hyperglycemia (Ugochukwu & Cobourne, 2003). After which STZ was intraperitoneally injected into 50 male mice aged 12 weeks, this caused a 20% mortality and the number of mice became (n=40). Mice were examined for blood glucose concentration using a glucometer (Accu-Check Performa) 48 h after STZ injection to induce hyperglycemia. The animals were diagnosed when their fasting blood glucose level was 7.8 mmol/l, as reported by the World Health Organization (Umar Imam *et al.*, 2019). Hyperglycemia mice were assigned to four groups with 10 mice per group (n=40): Streptozotocin control (STZ), 150, 300, and 500 mg/kg/body weight leaf extract of *C. nutans*. Methanolic leaf extract of the mice group was dispensed by oral gavage once daily for 28 days using distilled water as a carrier. The mice were given distilled water by oral gavage for both the control, normal chow diet (Normal), and STZ groups, ensuring that all animals received the same gavaging protocol, but without the methanolic extract. The animals were given no food for 12 h before being sacrificed, although they had unlimited access to water. The mice were given an intraperitoneal dose of ketamine 80 mg/kg/body weight and xylazine 10 mg/kg/body weight, and their blood was taken. Blood samples were taken and put into tubes through cardiac puncture using a one ml syringe and a 26-gauge needle. Weekly measurements of body weight and fasting blood glucose were taken using a laboratory weighing scale and glucometer (AccuChek Performa).

### Evaluation of Sperm Quality

Using a surgical microscissor, the left caudal epididymis was submerged in 2 ml of phosphate-buffered saline and sliced into roughly 200 pieces to liberate the spermatozoa from the epididymal tubules measured (Ngaha Njila *et al*., 2019). The epididymal semen suspension was right away kept at 37°C for future investigation. On a glass slide, a computer-assisted semen analysis (CASA, Hamilton Thorne Bioscience) tool was used to analyze the 5µl sperm suspension. The percentage of sperm mobility and progressive motility parameters were measured (Ngaha Njila *et al*., 2019).

### Analysis of Sperm Counts

One microliter of epididymal semen suspension was mixed with 199 microliters of formal saline to achieve a dilution factor of 1:1000. The total sperm concentration was determined by a Neubauer hemocytometer as recorded previously by Ngaha Njila *et al*. (2019).

### Morphology of Sperm

This test determines whether any morphologically aberrant spermatozoa are present and the frequency with which they occur. The sperm cells were evaluated for morphological abnormalities using the Wells and Awa stain. A drop of semen was dropped onto a clean hot glass slide, and a smear was made using a second slide stained with Wells and Awa stain. The dyed smear was air-dried before being inspected under a light microscope, an OLYMPUS MODEL CX21FS1. Head, mid-piece, and tail abnormalities were classified as spermatozoa abnormalities by Zemjanis (1970).

### Measurement of Antioxidant Enzymes Activity and Androgen Hormone in Serum

Sera samples were prepared by centrifuging blood samples at 4000 rpm for 10 minutes after collecting blood samples from mice, and sera were stored at -80°C.

Test for catalase activity, the procedure outlined by Aebi (1984) was used to measure serum catalase activity. Then, 0.2 ml of phosphate buffer was added to 2.8 ml of 30 mM H_2_O_2_ in a blind tube. The sample tube was then added 2.8 ml of 30 mM H_2_O_2_. The 0.2 ml of the enzyme was added to each of these tubes, and the tubes were vortexed to combine the contents. To determine the activity, the absorbances at 240 nm were tested twice at 30-s intervals.

Reduced glutathione (GSH) assay, the technique described by Beutler *et al.* (1963) was used to determine the GSH level. First, 800 ml of phosphate buffer was used to dilute 200 µl of serum, and the first absorbance (OD1) at 412 nm was determined. The second absorbance value (OD2) was then noted after 100 µl of Ellman’s reagent had been added to the same tube. The amount of GSH was calculated using the formula C/1000=(OD2-OD1)/13,600×E1×5/2×[(C: mmol/glutathione (mg/dl)]. mmol/ml.

Superoxide dismutase activities (SOD) assay, the approach suggested by Sun *et al*. (1988) was used to measure the serum SOD activity. This technique relies on measuring the optical density of the blue formazan dye at 560 nm, which is created when nitro blue tetrazolium reacts with superoxide radicals produced by xanthine and xanthine oxidase. By removing the superoxide radicals from the medium, the SOD present in the sample prevents the formazan process. Under assay conditions, one unit of SOD reduces the rate of nitro blue tetrazolium reduction by 50%.

Malondialdehyde (MDA) assay, the MDA was assessed as the production of its colored form with thiobarbituric acid (Aebi, 1984), one of the peroxidation products created by the interaction of fatty acids with free radicals. A tube containing 200 ml of the patient’s serum was filled with 800 ml of phosphate buffer, 25 ml of butylhydroxytoluene solution, and 500 ml of 30% trichloroacetic acid. After mixing, the tubes were kept on ice for two hours. One ml of each supernatant was then transferred to a fresh tube, to which 75 ml of ethylene diamine tetra acetic acid and 25 ml of thiobarbituric acid were then added. They were then centrifuged at 2000 rpm for 15 min. The tubes were combined and incubated for 15 minutes in a hot water bath. They were brought to room temperature, and a UV/Vis spectrophotometer was used to measure the absorbance at 532 nm.

In the total antioxidant capacity (TAOC) assay, the TAOC of serum was measured according to the method of Benzie & Strain (1999). Briefly, a working solution of FRAP (ferric reducing antioxidant power) was provided by mixing buffer acetate with TPTZ solution in HCl. After that, FeCl3 was added and mixed. 8 µL of serum and 240 µL of mentioned working solution were mixed and incubated for 10 min at room temperature. The optical density of samples was measured at 532 nm, and total antioxidant capacity was expressed as mmol/L.

According to the manufacturer’s instructions, a sandwich Enzyme-linked immunosorbent assay (ELISA) kit (CLOUD-CLONE CORP) was used to assess serum luteinizing hormones (LH) and testosterone concentrations in the hormonal analysis.

### Testicular Histology

The left and right testes were retrieved and fixed in Bouin’s solution once the sperm collection was completed. The samples were clipped into smaller pieces after two hours to allow the fixatives to penetrate better. Bouin’s held the samples for an additional 14 h. The tissue samples were also cleaned three times with 70% alcohol and stored overnight. The samples were then placed in cassettes in a basket before being processed by an automatic tissue processor. After that, stainless steel cassette molds were used to implant it. Once the wax had hardened, the blocks were cooled on an integrated cold plate and removed from the mold. After the extra wax was trimmed away, sections 4 to 5 µm thick were cut from the tissue block using a microtome. After that, the tissue slides were stained with hematoxylin, and eosin examined under light microscopy. The seminiferous tubules, morphology of various stages of spermatozoa development, and histological variations between treatment groups were all areas of interest.

### Statistical Analysis

The GraphPad Prism application was used to examine the data (Prism 7.0, GraphPad Software Inc., CA, USA). To express quantitative data, the mean and standard error of the mean (SEM) was utilized. The significance level between and among groups was then determined following the ANOVA one-way procedure and mean separation using Tukey’s honestly significant difference (HSD) at a 5% level of significance.

## RESULTS

### Body Weight and Blood Glucose

Body weight (g) and blood glucose data were collected weekly for each group, as shown in Table 1. One-way analysis of variance and the mean separation test revealed significant (5%) differences in baseline body weight between normal and diabetic mice [F (4,10)=3.66, *p*=0.04]. As a result, in week 4, the data revealed a clear difference between mouse groups [F (4,10)=56.3, *p*=0.01] in body weight, STZ mice had considerably lower body weight than normal mice, and mice were treated with methanol extract at various doses. In terms of baseline glucose (mmol/L) levels, as shown in Table 2, there was a statistical difference of 5% between the normal and diabetic groups [F (4,10)=17, *p*=0.001]. Meanwhile, in week 4, mice in the experimental group fed leaf extract from *C. nutans* at varied doses of 150, 300, and 500 milligrams/kilogram/ body weight resulted in a considerable drop in blood glucose levels compared to STZ mice [F (4,10)=17, *p*=0.001].

**Table 1:** Effects of *C. nutan*s leaf extracts on the body weight across male mice groups.

Weight (g)	Baseline	Week1	Week2	Week3	Week4
Normal	27±0.57^b^	28.7±0.66^b^	30±1.53^b^	32±0.88^ab^	33.7±0.88^ab^
STZ	36±0.57^a^	33.7±0.88^a^	30.3±0.88^b^	28±0.57^c^	26±1.53^c^
*C. nutans* 150 mg/kg	37±0.5^a^	35.7±0.88^a^	34.7±0.88^a^	31±1.15^b^	30.7±0.88^b^
*C. nutans* 300 mg/kg	37±1.15^a^	36±0.57^a^	35.3±0.66^a^	34.7±0.66^a^	33.3±0.33^ab^
*C. nutans* 500 mg/kg	36±0.57^a^	36±0.57^a^	35.7±0.33^a^	35.3±0.66^a^	34.3±0.88^a^
*p*-value	0.001	0.001	0.003	0.001	< 0.001

Values are stated in terms of mean and standard error (M ±SEM)^a, b, c, d, e^ Means followed by different letters in a column are statistically different (5% level) between groups by Tukey’s test (n=10). STZ= streptozotocin mice group, *C. nutans* = *Clinacanthus nutans*.

**Table 2:** Effects of *C. nutans* leaf extracts on glucose levels across male mice groups.

Parameters (%)	Normal	STZ	C. nutans 150 mg/kg	C. nutans 300 mg/kg	C. nutans 500 mg/kg	*p*-value
Tailless head	6.83±1.01^d^	30±15^a^	21.3±0.88^b^	15.3±0.88^c^	8.3±0.47^d^	<0.001
Headless tail	8.33±0.88^c^	29.3±1.45^a^	20.7±0.33^b^	11±1.15^c^	7.67±1.2^c^	<0.001
Bent tail	8.67±0.66^c^	30.3±2.41^a^	19±1.53^b^	9.67±1.2^c^	7.83±0.72^c^	<0.001
Looped tail	6.67±1.45^c^	21±1.53^a^	17±1.15^b^	8.47±0.86^c^	5.73±0.88^c^	<0.001
Rudimentary tail	7.67±0.66^c^	23.7±0.88^a^	16.7±1.2^b^	9.67±0.88^c^	6.67±0.98^c^	0.001
Mean sperm-abnormalities	33.3±0.88^d^	70.7±1.2^a^	57±2.31^b^	45.3±2.73^c^	33.3±1.2^d^	0.001

Values are stated in terms of mean and standard error (M ±SEM)

a,b,c,d,e Means followed by different superscript letters in a column are statistically different (5% level) between groups by Tukey’s test.

### Spermatozoa Abnormalities

A typical sperm’s head is spherical, and its tail is long. In aberrant sperm, head or tail malformations, such as a large or deformed head or a crooked or double tail, are prevalent. These defects may make it difficult for sperm to reach and penetrate an egg. This investigation (Table 3) revealed that the percentage of spermatozoa abnormalities was substantially (*p*=0.05) higher in STZ mice than in normal mice in most categories. After four weeks of feeding, hyperglycemia mice fed with methanol leaf extract at doses of 150, 300, and 500 mg/kg/body weight had considerably fewer normalities than STZ animals. At a dose of 500 mg/kg, leaf extract was demonstrated to cure mouse groups and strongly improve abnormalities.

**Table 3:** Effects of *C. nutans* leaf extracts on percentage spermatozoa abnormalities across male mice groups.

Glucose (mmol/L)	Baseline	Week1	Week2	Week3	Week4
Normal	4.47±0.08^b^	5.53±0.14^d^	4.53±0.14^e^	4.8±0.25^e^	4.47±0.26^e^
STZ	20.7±0.33^a^	24.5±0.28^a^	25.5±0.31^a^	25.7±0.90^a^	26±0.11^a^
*C. nutans* 150 mg/kg	20.4±0.35^a^	17.8±0.89^b^	16.6±1.03^b^	16.03±1.21^b^	14.2±0.17^b^
*C. nutans* 300 mg/kg	21.3±0.88^a^	17.5±1.01^b^	14±0.24^c^	13.1±0.71^c^	10.7±0.74^c^
*C. nutans* 500 mg/kg	22.3±1.18^a^	13.6±0.80^c^	11.3±0.40^d^	9.03±0.54^d^	7.3±0.92^d^
p-value	<0.01	0.001	<0.001	<0.001	<0.001

The mean and standard error (SEM) is used to express the data.

a, b, c, d Tukey’s test shows that means within a row with distinct superscript letters are significantly different (%5, ANOVA) between groups (n=10). STZ= streptozotocin mice group, *C. nutans* = *Clinacanthus nutans*.

### Sperm Motility, Progression and Count

**Figure 1:**
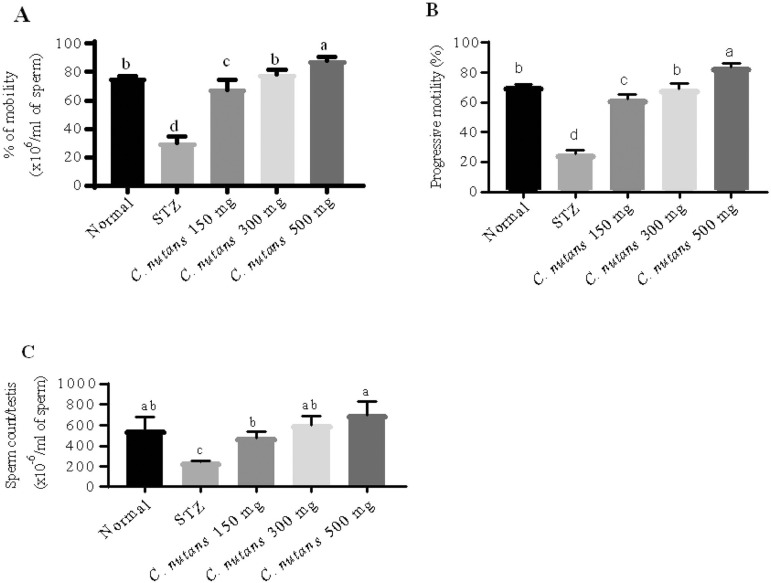
Effects of *C. nutans* leaf extracts on infertility across mice groups. (A) Percentage mobility, (B) progressive motility, and (C) sperm count in mice. STZ= streptozotocin mice group. Values are expressed as mean ± SEM. ^a,b,c,d^ The means with different letters differ statistically (5% level of probability) between groups by Tukey’s test (n=10). *C. nutans* = *Clinacanthus nutans*.

Figure 1A shows a statistical difference of 5% in sperm motility percentage across mouse groups [F (4,10) = 96.7, *p* = 0.001]. The STZ mice had a significantly lower motility level (30±2.5%, *p* = 0.001) than the normal mice (75.3±0.88%), according to a post hoc test. In comparison to STZ mice, hyperglycemia mice treated with *C. nutans* leaf extract at doses of 150 mg/kg (67.7±3.71%, *p* = 0.001), 300 mg/kg (78.3±1.67%, *p* = 0.001), and 500 mg/kg (88±1.53%, *p* = 0.001) showed a substantial increase in general motility sperm levels.

There was a statistical difference (5%) in progression levels among the mouse groups (Figure 1B) [F (4,10) = 182, *p* = 0.001]. In STZ mice, mean separation using the one-way ANOVA revealed a statistically significant decrease in progressive sperm level (25.7±1.2%, *p* = 0.001) as compared to normal mice (70.5±0.68%). In contrast to the STZ group, STZ treated with *C. nutans* leaf extract at doses of 150 mg/kg (62±2%, *p* = 0.001), 300 mg/kg (69±2%, *p* =0.001), and 500 mg/kg (83.3±1.67%, *p* = 0.001) showed a statistical increase in progression sperm level.

According to total sperm count influenced by *C. nutans* leaf extract fed diabetic mice (Figure 1C), one-way ANOVA revealed a significant difference in total sperm counting levels among the mice groups [F (4,10) = 96.6, *p*=0.002]. The STZ mice group (240±7.64, *p*=0.01) had a statistically significant (5 percent) lower total sperm counting level than the normal mice group (550±76.4). In comparison to STZ mice, meanwhile, STZ mice were treated with methnolic leaves extract of *C. nutans* at doses 150 mg/kg (480±35.1, *p*=0.04), 300 mg/kg (600±50, *p*=0.004), and 500 mg/kg (700±76.4, *p*=0.001) showed a significant increase in total sperm counts.

### Androgen Hormone

**Figure 2:**
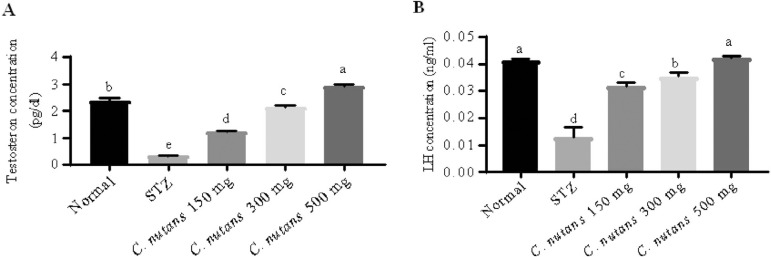
Effects of *C. nutans* leaf extracts on androgen hormone in male mice across treatment groups. (A) Testosterone concentration, (B) luteinizing hormone (LH) concentration, STZ= streptozotocin mice group. Values are expressed as mean ± SEM. ^a,b,c, d^, eMeans with different letters are statistically different (5% level of probability) between groups by Tukey’s test (n=10). *C. nutans* = *Clinacanthus nutans*.

After 4 weeks of methanolic leaves extract of *C. nutans* treatment, sex hormone levels increased. The testosterone hormone level was measured, and one-way analysis of variance revealed a statistical difference (*p*=0.05) in testosterone levels between the mouse groups [F (4,10)=611, *p*=0.001]. In comparison to the normal group (2.37±0.06pg/ml), the STZ group (0.31±0.01pg/ml, *p*=0.001) had a significantly lower testosterone level (0.31±0.01 pg/ml, *p*=0.001) (Figure 2A). Similarly, STZ mice given *C. nutans* leaf extract for 4 weeks at doses of 150 mg/kg (1.22±0.02pg/ml, *p*=0.001), 300 mg/kg (2.15±0.02pg/ml, *p*=0.001), and 500mg/kg (2.92±0.05pg/ml, *p*=0.001) demonstrated a statistically significant rise in testosterone levels when compared to STZ mice.

*A*fter 4 weeks of treatment with *C. nutans* leaf extract, luteinizing hormone (LH) hormone was identified in all mice groups, with a statistical difference (5 percent level) in LH levels [F (4,10)=110, *p*=0.001] (Figure 2B). The STZ group had a significantly lower LH level (0.013±0.02ng/ml, *p*=0.001) than the normal group (0.041±0.05ng/ml), according to Turkey’s post hoc test. In comparison to STZ mice, STZ mice treated with *C. nutans* leaf extract at doses of 150 mg/kg (0.031±0.08ng/ml, *p*=0.001), 300 mg/kg (0.035±0.08ng/ml, *p*=0.001), and 500 mg/kg (0.042±0.03 ng/ml, *p*=0.001) showed a substantial rise in LH hormone level.

### Anti-Oxidant Enzymes Activity

The anti-oxidants results are illustrated in Table 4. After 4 weeks of feeding, diabetic mice were treated with three different doses of leaf extract methanolic *C. nutans* (150, 300, and 500 milligram/kilogram/bodyweight) to show differences in treatment levels when compared to the STZ group of mice. Catalase (U/L), GSH (mmol/ml), MDA (mmol/L), SOD (U/ml), and TAOC (mmol/L) were all statistically different (5%) among the treatment groups of mice. When compared to the STZ group, mean separation using the Tukey test revealed a statistically greater level in the category of STZ treated with methanolic extract at doses of 150, 300, and 500mg/kg.

**Table 4:** Effects of *C. nutans* leaf extracts on antioxidant activity across male mice groups.

	Catalase (U/l)	GSH (mmol/ml)	MDA (mmol/l)	SOD (U/ml)	TAOC (mmol/l)
Normal	50.1±0.09^a^	7.5±0.15^a^	20.1±0.59^d^	38.1±0.15^a^	5.58.7±0.26^a^
STZ	20.2±0.58^d^	3.33±0.29^d^	46.9±0.95 ^a^	22.1±0.58^d^	2.73±0.23^d^
*C. nutans* 150 mg/kg	24.9±0.98^d^	4.7±0.20^c^	32.7.7±1.41^b^	27.3±0.58^c^	3.19±0.31^cd^
*C. nutans* 300 mg/kg	33.7±0.96^c^	5.37±0.23^c^	28.3±0.46^c^	32.5±0.43^bc^	3.86±0.07^cd^
*C. nutans* 500 mg/kg	43.7±0.64^b^	6.33±0.26^b^	23.1±0.7^d^	35.1±0.61^b^	4.57±0.32^b^
*p*-value	0.001	0.001	0.001	0.001	< 0.001

Values are stated in terms of mean and standard error (mean ±SEM)

a,b,c,d Means followed by different superscript letters in a column are statistically different (5% level) between groups by Tukey’s test (n=10). GSH = reduced glutathione, MDA= malondialehyde, SOD = superoxide dismutase, TAOC= total anti-oxidant capacity, STZ= streptozotocin mice group. *C. nutans* = *Clinacanthus nutans*.

### Testicular Histology

**Figure 3:**
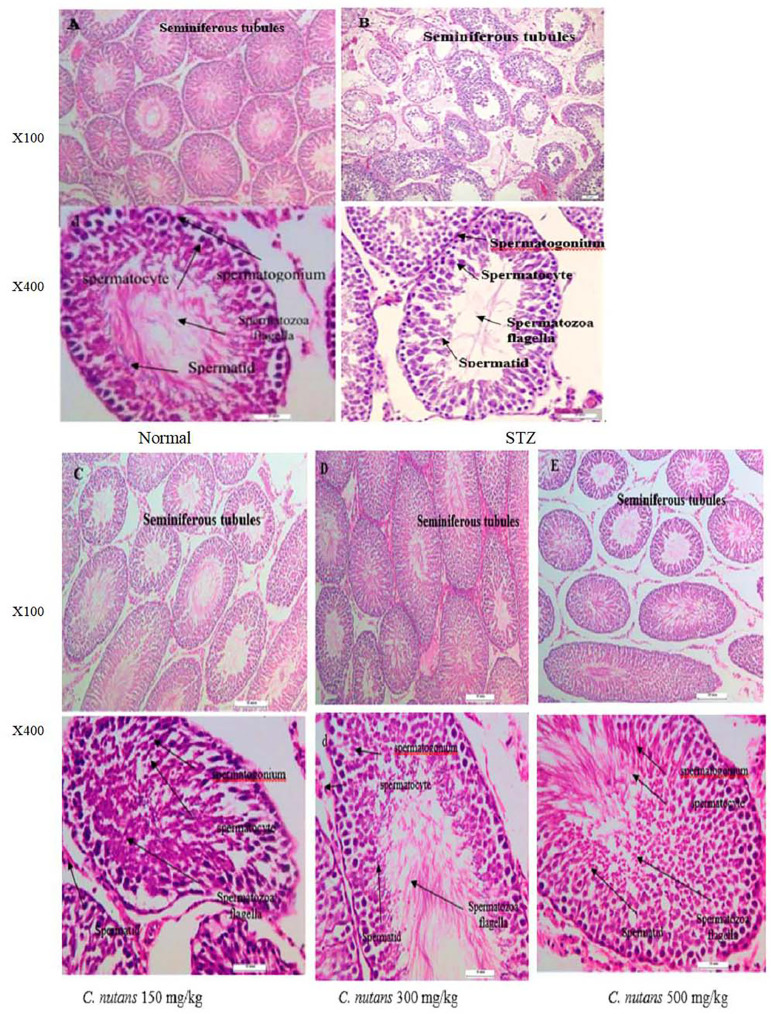
Histological structure of the testes across treatment groups (H&E staining, n=10) (A) Normal, mice treated with distilled water, (B) STZ (streptozotocin mice group), magnification X 200, and X400. (C) diabetic mice treated with leaf extract of *C. nutans* at dose 150 mg/kg body weight, (D) diabetic mice treated with leaf extract of *C. nutans* at dose 300 mg/kg body weight, (E) diabetic mice treated with leaf extract of *C. nutans* at dose 500 mg /kg body weight, magnification X 200, and X400. *C. nutans* = *Clinacanthus nutans*.

Within the convoluted seminiferous tubules, the normal control of the photomicrograph testes (Figure 3A) revealed normal histological features. The stratified epithelium of these convoluted seminiferous tubules was made up of two different cell populations: spermatogenic cells and Sertoli cells. All of the Leydig cells in the supporting tissues in the tubule interstitial spaces were evident. As a result (Figure 3B) in the STZ group, it was discovered that the cellular levels of spermatocytes and spermatids in seminiferous tubules had decreased, and the connections between cells had vanished. The thickness of the basement membrane was noted to be increasing, and the spaces between seminiferous tubules were extremely visible. Degeneration and necrosis of spermatogenic and interstitial (Leydig) cells were also seen. The testicular histology of STZ mice treated with *C. nutans* leaf extract at doses of 150, 300 and 500 mg/kg after 4 weeks revealed essentially intact seminiferous tubules, as well as a large number of active Leydig cells, indicating a higher possibility of fertility than diabetic mice. See Figure 3 (C, D, and E).

## DISCUSSION

Injecting STZ intraperitoneally into male mice causes problems not just with hyperglycemia but also with the male reproductive system and changes in sexual hormones. In the current investigation, a crude methanol extract of *C. nutans* was administered to male hyperglycemic mice at doses of 150, 300, and 500mg/kg body weight. Blood glucose levels were dramatically lowered as a result of the treatment, and sperm quality and antioxidant levels were also improved. *Clinacanthus nutans,* according to the author, improve the testicular structure. Comprehensive functional studies of *C. nutans* are expected to pave the way for its use in metabolic illness treatment. People employ plant extracts for a variety of ailments because of the high cost and ineffectiveness of pharmaceuticals.

In terms of body weight, hyperglycemic mice were given a high dose of 300 and 500 mg/kg body weight of *C. nutans* leaf extract did not significantly lose weight when compared to their initial weight. This is more likely because the extract prevented fat and protein from breaking down to produce glucose. Depending on their starting weights, mice of the same age lost weight in different ways. In experimental models of hyperglycemia, it’s crucial to keep an eye on the connection between the course of diabetes and weight loss in order to prevent excessive weight loss (>10 percent) and drop-in animal survival (Dekel *et al.*, 2009). In another trial, a dose of 200mg STZ per kg of body weight likewise resulted in a 20% weight loss; however, when the dose was reduced to 150 mg STZ per kg, the weight loss was stopped, and when it was further decreased to 100mg/kg, weights grew as expected for 8-week-old ICR mice (Hayashi *et al.*, 2006). The catabolism of proteins and body fat would have been reduced, which would have reduced weight loss caused by gluconeogenesis if the extract had been suppressed. In type 2 diabetes rats, gluconeogenesis is increased because the body’s cells do not utilize enough glucose for metabolism (Umar Imam *et al*., 2019). For many of the problems related to human diabetes, the high-fat model is often regarded as the most accurate (Furman, 2015).

In this study, it was revealed that diabetic mice given a metabolic extract of *C. nutans* had decreased fasting blood glucose levels. The activity of multiple phenolic compounds found in the extracts and fractions of *C. nutans*, such as caffeic acid, gallic acid, p-coumaric acid, chlorogenic acid, ferulic acid, vanillic acid, protocatechuic acid (Sarega *et al.*, 2016), 6-C-glucosyl-8-arabinosyle apigenin, or arabinosyl glucosyl apigenin (Abdulwahid-Kurdi, 2019), could be responsible for blood glucose (Antora & Salleh, 2017). Apart from the aforementioned, flavonoids, sterols, alkaloids, saponins, tannin, polyphenols, alkaloids, carbohydrates, terpenoids, steroids, coumaric, and ferulic acids may all have a role in the bioactive antidiabetic activity of these herbal plants (Abdullah & Kasim, 2017). These antioxidant chemicals found in *C. nutans* leaf extract (Sarega *et al.*, 2016) have been shown to repair pancreatic islets in streptozotocin-induced hyperglycemia mice, enhancing insulin output (Vessal *et al.*, 2003). Low LH levels and, as a result, testicular dysfunction is caused by alterations in Leydig cells and the pituitary-testicular axis caused by high blood glucose (Arikawe *et al.*, 2012).

This study showed a dose-dependent decrease in sperm abnormalities in diabetic mice treated with *C. nutans* leaf extract at doses of 120, 300, and 500 mg/kg/body weight compared to STZ animals. The spermatozoa movement parameters were objectively quantified using computer-assisted semen analysis (CASA). For each individual cell, this technology delivers exact, precise, and relevant information. Similarly, the results of this experiment are comparable to those reported by Oladipupo & Abiodun (2020), who found a dose-dependent substantial increase in sperm motility and sperm count after administering varied dosages of *Cyperus esculentus* aqueous extract of *Cyperus esculentus* alone for 29 days. The epididymis, where the sperm membrane matures, may alter sperm motility. MECN leaves are self-evidently involved in spermatozoa maturation during transit through the epididymis, and the quality of the extract is dictated by the dose used. In rats, inducing hyperglycemia resulted in a substantial reduction in sperm count, viability, motility, and normal morphology, according to Roshankhah *et al.* (2017), which is similar to the results of our investigation.

The epididymis is where the sperm membrane matures and, according to Murono *et al.* (2006), is linked to sperm motility. As a result, it’s apparent that the methanolic extract of *C. nutans* leaves will alter spermatozoa maturation throughout their transit into the epididymis and that the dose will affect sperm quality. The production of normal and mature sperm is essential for optimum male fertility. The anterior pituitary secretes FSH and LH, which are responsible for the creation of sperm cells (spermatozoa) and testosterone in the testis (Steinberger, 1971). Ghorbani *et al*. (2014) found that eating walnuts as part of a diabetic diet reduced serum glucose levels and improved sex hormone secretion in male diabetics. Furthermore, diabetes has been demonstrated to affect the male hypothalamus-pituitary axis of the gonad, resulting in a considerable drop in testosterone (Maneesh *et al.*, 2006). Changes in androgenic hormones like luteinizing hormone (LH) and testosterone levels have also been associated with diabetes (Arikawe *et al.*, 2012).

In contrast to the control group, Balamurugan *et al.* (2014) found that treatment with an enhanced antioxidant enzyme activity (SOD, catalase, and glutathione) decreased aberrant spermatozoa and increased sperm motility and count with an ethanolic extract of *Melastoma malabathricum* (which contains saponin and tannin). According to Ismail & Radzi (2013), date fruit extracts have been demonstrated to have antioxidant capabilities against cypermethrin-induced oxidative stress because they include antioxidants such as coumaric and ferulic acids, which may result in increased motility. Spermatogenesis is a complicated process that can be influenced by a variety of variables that might lead to a reduction in male fertility. One reason is oxidative stress, which is generated by an imbalance between the antioxidant and oxidant systems and results in the creation of reactive oxygen species (Ismail & Radzi, 2013).

As indicated by hyperglycemia, mice with hyperglycemia have considerably lower anti-oxidant levels than mice treated with *C. nutans* leaf extracts in this study. Because of their oxidative stress properties, reactive oxygen species have pathogenic effects and cause organ malfunction when the number of oxides exceeds the number of antioxidants (Sikka *et al.*, 1995). According to animal research, when reactive oxygen species levels are high, semen’s antioxidant ability decreases, which might cause reproductive issues (Sikka *et al.*, 1995). Green tea may alleviate inflammation in addition to its antioxidant benefits, prevent DNA breakage, and increase sperm motility and viability (Sukcharoen *et al.*, 1995). Flavonoids are well-known antioxidants that can benefit animals with oxidative stress-induced testicular impairments (Asadi *et al.*, 2017). It also encourages androgenesis in the testes, which is required for testicular differentiation, integrity, and steroidogenic activity (Dafaalla *et al.*, 2016). Ekaluo *et al.* (2015) investigated the effects of an aqueous extract of *Cyperus esculentus* on sperm parameters and testosterone levels in male albino rats. The researchers discovered that the aqueous extract of *Cyperus esculentus* has antioxidant and androgenic properties, as well as the ability to boost sperm quality and testosterone levels. As a result, they speculated that an aqueous extract of *Cyperus esculentus* could be used to boost fertility while lowering sperm and reproductive toxicity (Ekaluo *et al.*, 2015). The STZ-induced diabetic mice’s testis showed frequent aberrant architectural changes in the seminiferous tubule lined by spermatogenic cell series, Sertoli cells, and Leydig cells, which resulted in a reduction in plasma testosterone levels (Ricci *et al.*, 2009). In comparison to STZ mice, hyperglycemia mice treated with *C. nutans* leaf extract at doses of 300 and 500 mg/kg increased spermatogenesis, as evidenced by a considerable increase in the number of spermatids, spermatogonia, spermatocytes, and Sertoli cells. Furthermore, the increase in blood testosterone levels following leaf extract of *C. nutans* treatment can be attributable to an increase in the number of interstitial Leydig cells on the one hand and increased LH secretion on the other. After 4 weeks, a photomicrograph of the testicular tissue of diabetic mice treated with methanolic extract at various doses. The majority of seminiferous tubules have normal structure, and just a few have necrotic alterations (Figure 3C).

*Clinacanthus nutans,* according to Abdulwahid-Kurdi (2019), can be used as a good source of selenium. Furthermore, the role of selenium in rat spermatogenesis appears to be unique, and it cannot be replaced by vitamin E or any other antioxidant (Wu *et al.*, 1973). Leaf extracts of *C. nutans* at doses of 150, 300, and 500 mg/kg/body weight were selected following a review of the previous study as well as to discover the best dose among these three for hyperglycemia and reproductive difficulties. The body responses of mice vary depending on the type of extract, dose, time, and method of administration, as well as environmental conditions and their effect on the phenolic profile in the leaf (Ekpenyong *et al*., 2014). Thus, it is crucial to consider the factors that influence chemical variability in plant species. Among them are physiologic changes, environmental factors, regional variances, genetic factors, the quantity of plant material and/or space needed, and the requirements for manual work (Figueiredo *et al*., 2008).

In conclusion, *Clinacanthus nutans* extract has certain androgenic effects, including the capacity to preserve sperm against diabetes-related morphological abnormalities. The leaves of *C. nutans* have antioxidant properties, which could be one of the reasons for their positive impact on spermatic parameters. It is reasonable to conclude that *C. nutans*’s methanolic leaf extract affects the fertility of male hyperglycemia mice and that it could be used to correct sperm morphological problems brought on by particular drugs, like streptozotocin. The medicine may act on the pituitary gland due to increased sperm motility in the cauda epididymis, increasing the primary spermatogenesis hormone in the treated group. As a result, high blood sugar levels in male mice may be treated with *C. nutans* leaf extract to enhance antioxidants and sperm quality. Further studies will be published in the near future.

## Data Availability

The data used in this work are provided to the corresponding author upon justifiable request.
